# Breakthroughs in Medicinal Chemistry: New Targets and Mechanisms, New Drugs, New Hopes–2

**DOI:** 10.3390/molecules23010065

**Published:** 2017-12-28

**Authors:** Diego Muñoz-Torrero, Arduino A. Mangoni, Hong Liu, Christopher Hulme, Jarkko Rautio, Rafik Karaman, Maria Emília de Sousa, Katalin Prokai-Tatrai, Jean-Marc Sabatier, Carlo Siciliano, F. Javier Luque, George Kokotos, Rino Ragno, Simona Collina, Catherine Guillou, Michael Gütschow, Luigi A. Agrofoglio

**Affiliations:** 1 Laboratory of Pharmaceutical Chemistry, Faculty of Pharmacy and Food Sciences, and Institute of Biomedicine (IBUB), University of Barcelona, Av. Joan XXIII, 27-31, E-08028 Barcelona, Spain; 2 Department of Clinical Pharmacology, College of Medicine and Public Health, Flinders University and Flinders Medical Centre, Bedford Park, SA 5042, Australia; arduino.mangoni@flinders.edu.au; 3 State Key Laboratory of Drug Research, Key Laboratory of Receptor Research, Shanghai Institute of Materia Medica, Chinese Academy of Sciences, 555 Zu Chong Zhi Road, Shanghai 201203, China; hliu@simm.ac.cn; 4 Department of Pharmacology and Toxicology, and Department of Chemistry and Biochemistry, College of Pharmacy, The University of Arizona, Biological Sciences West Room 351, 1041 East Lowell Street, Tucson, AZ 85721, USA; hulme@pharmacy.arizona.edu; 5 School of Pharmacy, Faculty of Health Sciences, University of Eastern Finland, P.O. Box 1627, FI-70211 Kuopio, Finland; jarkko.rautio@uef.fi; 6 Pharmaceutical & Medicinal Chemistry Department, Faculty of Pharmacy, Al-Quds University, POB 20002 Jerusalem, Palestine; dr_karaman@yahoo.com; 7 Department of Sciences, University of Basilicata, Viadell’Ateneo Lucano 10, 85100 Potenza, Italy; 8 Laboratório de Química Orgânica e Farmacêutica, Departamento de Ciências Químicas, Faculdade de Farmácia, Universidade do Porto, Rua Jorge Viterbo Ferreira 228, 4050-313 Porto, Portugal; esousa@ff.up.pt; 9 Interdisciplinar de Investigação Marinha e Ambiental (CIIMAR/CIMAR), Universidade do Porto, Terminal de Cruzeiros do Porto de Leixões, Avenida General Norton de Matos, S/N 4450-208 Matosinhos, Portugal; 10 Center for Neuroscience Discovery, Department of Pharmaceutical Sciences, University of North Texas, Health Science Center, 3500 Camp Bowie Blvd, Fort Worth, TX 76107, USA; katalin.prokai@unthsc.edu; 11 Laboratory INSERM UMR 1097, Aix-Marseille University, 163, Parc Scientifique et Technologique de Luminy, Avenue de Luminy, Bâtiment TPR2, Case 939, 13288 Marseille, France; sabatier.jm1@libertysurf.fr; 12 Department of Pharmacy, Health and Nutritional Sciences, University of Calabria, I-87036 Arcavacata di Rende, Italy; carlo.siciliano@unical.it; 13 Department of Nutrition, Food Science, and Gastronomy and Institute of Biomedicine, University of Barcelona, Av. Prat de la Riba 171, 08921 Santa Coloma de Gramenet, Spain; fjluque@ub.edu; 14 Laboratory of Organic Chemistry, Department of Chemistry, National and Kapodistrian University of Athens, Panepistimiopolis, 15771 Athens, Greece; gkokotos@chem.uoa.gr; 15 Rome Center for Molecular Design, Dipartimento di Chimica e Tecnologie del Farmaco, Sapienza Università di Roma, P. le A. Moro 5, 00185 Roma, Italy; rino.ragno@uniroma1.it; 16 Department of Drug Sciences, Medicinal Chemistry and Pharmaceutical Technology Section, Centre for Health Technologies (CHT), University of Pavia, Viale Taramelli 12, 27100 Pavia, Italy; simona.collina@unipv.it; 17 Institut de Chimie des Substances Naturelles, CNRS UPR 2301, Unversité de Paris-Saclay, 91198 Gif-sur-Yvette, France; catherine.guillou@cnrs.fr; 18 Pharmaceutical Institute, University of Bonn, An der Immenburg 4, 53115 Bonn, Germany; guetschow@uni-bonn.de; 19 ICOA UMR CNRS 6005, Universite d’Orleans, Rue de Chartres, 45067 Orleans CEDEX 2, France; luigi.agrofoglio@univ-orleans.fr

## 1. Introduction

*Breakthroughs in Medicinal Chemistry: New Targets and Mechanisms, New Drugs, New Hopes* is a series of *Editorials*, which are published on a biannual basis by the Editorial Board of the Medicinal Chemistry section of the journal *Molecules*. In these *Editorials*, we highlight, in brief reports of about one hundred words, a number of recently published articles that describe crucial findings such as the discovery of novel drug targets and mechanisms of action or novel classes of drugs, and may inspire future medicinal chemistry endeavors devoted to address prime unmet medical needs.

## 2. An Allosteric Inhibitor with the Most Complex Mode of Action to Combat Tuberculosis

### Highlighted by Diego Muñoz-Torrero

New antitubercular agents with novel mechanisms of action are needed to cope with the steady rise in multidrug resistance. Phenotypic screening of a diversity-oriented synthetic library has led to the identification of (2*R*,3*S*,4*R*)-3-[2′-fluoro-(1,1′-biphenyl)-4-yl]-4-(hydroxymethyl) azetidine-2-carbonitrile (BRD4592), which kills *Mycobacterium tuberculosis* (Mtb) from a diverse panel of clinical isolates, including drug-resistant strains [1]. BRD4592 hits the essential metabolic enzyme tryptophan synthase (TrpAB), which contains two α and two β subunits that catalyze the penultimate and last steps of l-tryptophan biosynthesis. BRD4592 is an allosteric inhibitor of α and β subunits. By binding at the α-β interface along the tunnel that connects both subunits, BRD4592 blocks shuttling between the two active sites of indole produced at the α subunit to be converted into l-tryptophan at the β subunits. Moreover, it stabilizes crucial enzyme states, including the enzyme-product complex, thereby preventing catalytic cycling. The unique multicomponent mode of action of BRD4592 will hopefully inspire the design of a new generation of highly potent allosteric Mtb TrpAB inhibitors.

## 3. Inhaled Selective Indazole Ether-Based Glucocorticoid Receptor Modulators: A Novel Treatment Strategy for Asthma and Chronic Obstructive Pulmonary Disease?

### Highlighted by Arduino A. Mangoni

The global health burden of asthma and chronic obstructive pulmonary disease (COPD) is increasing despite preventive strategies and available inhaled anti-inflammatory therapies. The latter, particularly glucocorticoids, are associated with local and systemic adverse effects. Hemmerling et al., following the discovery of indazole ethers as potent nonsteroidal glucocorticoid receptor modulators, implemented a soft-drug strategy with further optimization that led to a lead compound, 3-(5-((1*R*,2*S*)-2-(2,2-difluoropropanamido)-1-(2,3-dihydrobenzo-[*b*][1,4]dioxin-6-yl) propoxy)-1*H*-indazol-1-yl)-*N*-((*R*)-tetrahydrofuran-3-yl)benzamide, with high selectivity for the progesterone receptor [2]. In pharmacokinetic studies in rats, this compound showed, when administered by inhalation, slow dissolution, low volume of distribution, and relatively high lung retention. In a rat model of Sephadex-induced airway inflammation, this compound effectively inhibited lung edema, particularly when pre-dosed 9 h prior to challenge. Pending further pharmacodynamic/pharmacokinetic characterization and optimization, this study might pave the way for the development of a new class of drugs for asthma and COPD. 

## 4. The Advancement of Developing Potent Proteolysis-Targeting Chimeras (PROTACs) in Anticancer Drug Discovery 

### Highlighted by Hong Liu

PROTACs are a novel class of heterobifunctional molecules designed to recruit a target protein to an E3 ubiquitin ligase complex, enabling following poly-ubiquitination and degradation of the target. PROTACs are characterized by inhibiting the activity of specific proteins that drive tumor growth by targeted degradation, thus they may be useful in the drug-resistance of cancer. Recently, PROTACs designed to degrade the BRD4 protein have been reported by several labs. In 2017, Alessio Ciulli et al. [3] revealed the first ternary crystal structure of the PROTAC molecule (BRD4 degrader MZ1) with the target protein (BRD4 bromodomain) and E3 ubiquitin ligase (human VHL), which provides structural insights into how PROTACs induce target-specific protein–protein interactions of the target protein with an E3 ligase to form the isoform-specific ternary complexes for selective degradation. At the same time, Shaomeng Wang et al. [4] reported a new class of PROTAC inducing BRD4 degradation, which structurally combined BET4 inhibitor HJB97 with thalidomide as the cereblon ligand. The most potent inhibitor (BETd-260) of this series effectively degrades BRD4 protein with an IC_50_ value of 30 pM in the RS4;11 leukemia cell line with a good selectivity over BRD2 and BRD3, and achieves extremely excellent RS4;11 cell-growth inhibitory activity (IC_50_ = 51 pM), and shows remarkable in vivo tumor suppression activity against RS4;11 xenograft tumors with 5 mg/kg IV dose without apparent body weight change. Although now the poor oral PK properties appear to be the biggest potential limitation of PROTACs, all these data above indicate that PROTACs are a most promising strategy of targeted protein degradation, thus making it a potential targeted therapeutic method for cancer. 

## 5. Dyrk1 Inhibition Improves Alzheimer’s Disease-Like Pathology

### Highlighted by Christopher Hulme

The dual-specificity tyrosine phosphorylation-regulated kinase-1A (Dyrk1a) is a protein kinase that phosphorylates the amyloid precursor protein (APP) and tau and thus represents a link between two key proteins involved in AD pathogenesis. As such, inhibition of Dyrk1a and restoration of its activity to normal levels in AD patients is postulated to alleviate both major aggregates in AD—amyloid plaques and insoluble tau aggregates—a unique, and the only pleiotropic targeting approach in the aggregation field. Results presented by Oddo et al. reveal the discovery of the first selective, brain penetrant Dyrk1a inhibitor that enticingly significantly reduces both Aβ42 levels and insoluble tau aggregates, simultaneously improving cognition, in well-tolerated fashion, during an 8-week study in 10 month old 3xTg-AD female mice [5].

## 6. The ProTide Prodrug Technology from the Concept to the Clinic

### Highlighted by Jarkko Rautio 

The ProTide prodrug technology that was discovered by Prof. Chris McGuigan is an efficient strategy to enable intracellular delivery of nucleoside analogue monophosphates and monophosphonates [6]. Nucleoside analogs, which have shown to be effective against cancer and various infections, must undergo the stepwise addition of phosphate groups mediated by cellular kinases to form the corresponding active nucleoside triphosphates. To circumvent the first rate-limiting phosphorylation step, nucleoside analogs are frequently dosed as their monophosphorylated forms or are structured to contain a phosphonate form. In ProTide strategy, the hydroxyl groups of the monophosphate or monophosphonate groups are masked with an aromatic and an amino acid ester promoieties. Lipophilic prodrugs are efficiently delivered to the cytosol of hepatocytes where the corresponding active triphosphate forms are formed after several consecutive enzymatic cleavage and addition reactions, respectively. To date, two ProTide prodrugs, tenofovir alafenamide and sofosbuvir, have been approved by the FDA. In addition, several other ProTide prodrugs have been tested in clinical trials. Those include GS-5734, which is being developed as a treatment of Ebola and other emerging viruses, and is engaged in Phase II clinical trials. *This review thoroughly summarizes the applicability of the ProTide prodrug technology in the improvement of drug delivery and efficacy.*

## 7. Understanding and Sensitizing Density-Dependent Persistence to Quinolone Antibiotics

### Highlighted by Rafik Karaman

A novel approach to make bacteria more sensitive to the antibacterial agent quinolones, which are utilized in the treatment of infections caused by *Escherichia coli* and *Staphylococcus aureus*, has been discovered by MIT researchers. The new approach has succeeded to overcome major limitations of these antibacterial agents which involve failures when used to treat infections characterized with a very high density of bacteria [7]. 

In 2011, Collins’s group found that the ability of the aminoglycoside antibiotics to kill drug-resistant bacteria can be increased by delivering a sugar along with the drug. The sugar helps to accelerate the metabolism of the bacteria, thus enabling the bacteria to undergo cell death in response to the DNA damage caused by the drug.

Due to side effects associated with the use of aminoglycosides, the group switched their research towards boosting the effectiveness of quinolones. With quinolones, the researchers found that a combination of a sugar and a terminal electron acceptor is a must to achieve the desired effect [7]. 

It is believed that this new approach has great potential to increase the effectiveness of existing antibacterials to kill bacteria that cause chronic infections.

## 8. Durable Antibiotic to Overcome Vancomycin Resistance with Synergistic Mechanisms 

### Highlighted by Maria Emília de Sousa

The spread of resistant bacteria, leading to untreatable infections, is a global threat. Several approaches to overcome antibacterial drug resistance have disclosed new antibiotics acting by multiple mechanisms. The research group of D. Boger [8] from the Scripps Research Institute in San Diego, California, described an approach to design durable antibiotics targeting vancomycin-resistant Enterococci (VRE), endowed with multiple synergistic mechanisms of action. Inspired by the nonselective membrane disruption induced by quaternary ammonium salts, the group introduced an alternative C-terminal peripheral modification at vancomycin analogs that induced membrane permeability without membrane depolarization or cell wall lysis. This approach culminated in an antibiotic 25,000-fold more potent than vancomycin against VRE with three independent mechanisms of action: membrane permeability induced by the C1 quaternary ammonium salt, inhibition of bacterial cell wall biosynthesis by direct transglycosylase inhibition caused by the (4-chlorobiphenyl)methyl modification and only one mechanism was dependent on d-Ala-d-Ala/d-Ala-d-Lac binding, the molecular basis of resistance to vancomycin. Moreover, this new vancomycin analog reduced susceptibility to resistance and represents a remarkable accomplishment in the total synthesis of glycopeptides. A remarkable example of multiple synergistic medicinal chemistry approaches!

## 9. Brain-Selective Estrogen Therapy via a Unique Bioprecursor Prodrug Approach

### Highlighted by Katalin Prokai-Tatrai

In order to exploit the vast array of beneficial effects of 17β-estradiol (E2) in the brain, brain-selective delivery of the hormone is needed to ensure therapeutic safety and efficacy. This elusive goal may now finally be accomplished in clinical settings by discovering that 10β,17β-dihydroxyestra-1,4-dien-3-one (DHED) is rapidly converted to E2 in the brain with extraordinary selectivity compared to the rest in the body. Therefore, DHED is a brain-selective bioprecursor prodrug of E2 that utilizes an NAD(P)H-dependent and brain-specific enzyme-catalyzed process to produce the hormone within the brain [9]. DHED is obtained by a simple stereoselective oxidation of the phenolic A-ring of E2, resulting in significantly improved physicochemical properties in the context of brain uptake from the circulation. With a series of in vitro and in vivo studies in preclinical animal models, the tremendous translational potential of DHED owing to its unprecedented and distinguishing feature in terms of brain-selective delivery of the parent hormone have been convincingly shown [9]. 

## 10. New Avenues in the Bacterial Mass Bioproduction of Molecules with ‘Improved’ Characteristics

### Highlighted by Jean-Marc Sabatier

The work by Chiang and collaborators [10] describes for the first time the biotransformation of a crude extract of soy isoflavones containing antioxidant glycosides daidzin and genistin, using the bacterium *Escherichia coli* expressing the recombinant tyrosinase from *Bacillus megaterium*. Interestingly, the main products of biotransformation were characterized as 3′-hydroxyisoflavone glycosides (i.e., 3′-hydroxydaidzin and 3′-hydroxygenistin) and showed respectively 120- and 72-fold superior 2,2-diphenyl-1-picrylhydrazyl free radical scavenging activities compared to their daidzin and genistin precursors. Therefore, apart from reporting on the successful bioproduction (and potential antioxidant/reductant applications) of two 3′-hydroxyisoflavone glycosides, this innovative work potentially paves the way to the mass bioproduction of other interesting compounds/derivatives with some 'optimized' functional characteristics.

## 11. Fighting the War against Type 2 Diabetes

### Highlighted by Carlo Siciliano

Compounds stimulating insulin production represent promising candidates for the treatment of type 2 diabetes, a worldwide cause of morbidity and mortality. Today, type 2 diabetes is controlled by a limited number of drugs and/or insulin injections, and no really effective medical treatments exist to defeat this pathology. Stanford et al. [11] gave a new hope publishing the full characterization of the relevant role played by low molecular weight protein tyrosine phosphatase (LMPTP) in promoting insulin resistance and type 2 diabetes. After an exhaustive SAR evaluation, the first bioavailable small molecule LMPTP inhibitor, namely the *N*,*N*-diethyl-3-[3-(piperidin-1-yl)propylamino]-4-(quinolin-2-yl)benzamide, was discovered. This compound demonstrated extraordinary potency and selectivity in modulating the LMPTP activity in vivo. The mechanism of LMPTP inhibition was deeply investigated, enlightening the effects of chemical inhibition of the enzyme. This approach was remarkably effective in improving glucose tolerance, reversing high-fat diet-induced diabetes without toxicity. Since LMPTP is known to promote heart failure and tumor growth, the newly discovered LMPTP inhibitor is a flag point and a game-changer for the future research addressed to the development of new drugs against human diabetes.

## 12. Exploring the Stability of Ligand Binding Modes to Proteins by Molecular Dynamics Simulations: A Cross-Docking Study

### Highlighted by F. Javier Luque

Docking is a fundamental approach for virtual screening of ligands and prediction of the binding mode to their target. However, several poses can be generated for a given ligand and identification of the correct binding mode is still challenging. Following a previous study [12], Liu and Kokubo [13] have examined the suitability of Molecular Dynamics (MD) simulations to discriminate between correct and decoy poses. Assuming the reliability of the classical force field, the underlying hypothesis is that a correct pose should be stable enough to maintain the initial pose more steadily than decoy poses. Accordingly, they have compared the suitability of a computational strategy that examines the structural integrity of the binding mode for a series of independent MD simulation runs. This is performed for three distinct target proteins (thrombin, heat shock protein 90-alpha, and cyclin-dependent kinase 2), each containing docking ligands in the pose closest to the X-ray crystallographic arrangement, and in two decoy poses chosen according to score ranking and geometrical deviation from the crystal structure. Keeping in mind the reduced computational cost of these computations, analysis of multiple MD equilibrium simulations may prove to be valuable for eliminating decoy poses that cannot be distinguished exclusively by docking scores or through complementary (i.e., pharmacophore) data, while being much less expensive than more accurate and computationally demanding free energy calculations.

## 13. Elovanoids: A Novel Class of Lipid Mediators Necessary for Photoreceptor Cell Integrity

### Highlighted by George Kokotos

Lipids are the major constituents of cell membranes and the most efficient source of energy. However, it is now fully recognized that lipids also function directly as signaling mediators. Several families of bioactive lipids regulate a plethora of cell functions and play prominent roles in immune regulation, inflammation and maintenance of homeostasis. Recently, oxygenated products derived from eicosapentaenoic acid (EPA) and docosahexaenoic acid (DHA) have attracted special attention as a new class of pro-resolving lipid mediators including resolvins and protectins [14]. The identification of novel bioactive lipids and the understanding of their function are of fundamental importance. Bazan’s group reported a novel class of lipid mediators, called elovanoids (ELVs), which are necessary for neuroprotective signaling for photoreceptor cell integrity [15]. Very long chain (≥C28) polyunsaturated fatty acids including n-3 (VLC-PUFAs,n-3) are generated from the DHA- or EPA-derived 26 carbon fatty acid by the action of elongase ELOVL4. ELVs are oxygenated derivatives of VLC-PUFAs,n-3. The structures, stereochemistry and bioactivity of ELVs were determined using synthetic compounds generated by stereo-controlled organic synthesis. These findings unveil a novel autocrine/paracrine pro-homeostatic retinal pigment epithelial cell signaling that aims to sustain photoreceptor cell integrity and reveal potential therapeutic targets for retinal degenerations.

## 14. Identification and Structural Characterization of First-in-Class Small Molecule Sirtuin 6 Activators

### Highlighted by Rino Ragno 

Sirtuins (Sirts) are protein deacylases that regulate metabolism and stress responses, and are implicated in various aging-related diseases, including cancer. Small molecule activators for the human Sirt1-7 have been proposed as chemical tools and potential therapeutics, but, with the only exception of Sirt1 activators, drug-like activators for Sirt2-7 are still missing. In particular, Sirt6 has been reported to contribute in modulating metabolic adaptations, DNA homeostasis, life-span extension and cancer growth and hence pharmacological Sirt6 modulation (inhibition and activation) is thus considered a potential treatment for cancer and aging-related pathological conditions. Recently, You et al. [16] reported a brief series of pyrrolo[1,2-*a*]quinoxaline derivatives as the first synthetic Sirt6 activators, endowed with a direct, substrate-independent binding capability to the enzyme catalytic core, and a strong activation of Sirt6-dependent deacetylation of peptide and protein substrates. Extensive structure-based investigation by means of chemical synthesis coupled with three-dimensional structure determination of several Sirt6/activator complexes revealed that the compounds bind to a specific acyl channel pocket highlighting key molecular interactions that provide the structural basis for further development of Sirt6 activators as both tools and therapeutics. The report by You et al. [16] shed light on the role of Sirt6 and opens new frontiers in the complicated epigenetics’ labyrinth.

## 15. Aquaporin-4: A Promising Target for Treating Neurological Disorders

### Highlighted by Simona Collina

Aquaporins (AQs) are a family of transmembrane proteins which manage water transport across biological membranes and water homeostasis; therefore, they can be pursued as therapeutic targets and the identification of related modulatory drugs is of high interest for medicinal chemists. In their recent paper, Verkman and colleagues discuss the potential of aquaporin-4 (AQ4) as drug target and outline the therapeutic potential of its modulators for treating neurological disorders related to water homeostasis imbalance, such as cerebral edema. Although drugging the AQs class still represents a challenging task, the availability of AQP4 crystal structure and the advances in screening approaches should enhance the chances for the successful identification of small molecules acting as AQP4 modulators. This interesting article will inspire new challenging drug discovery programs [17].

## 16. Copper–Alkyne Complexation Responsible for the Nucleolar Localization of Quadruplex Nucleic Acid Drugs Labeled by Click Reactions

### Highlighted by Catherine Guillou

Copper (I)-catalysed 1,3-dipolar azide-alkyne cycloaddition (CuAAC) has been widely used for labeling biological targets in cells. Copper-free cycloaddition promoted by strained cyclooctynes (strain-promoted azide–alkyne cycloaddition, SPAAC) is a major breakthrough in live cell imaging. G-Quadruplex(es) (G4) are noncanonical nucleic-acid structures found in guanine-rich sequences. They can be targeted with small molecules (G4 ligands) acting as reporters, for tracking both in vitro and in cells. The group of M.P. Teulade–Fichou reported that the results are different when using CuAAC or SPAAC methodologies. They demonstrated the need for great care when using CuAAC to localize drugs in cells, and show that SPAAC gives results that are more consistent between fixed and live cells [18].

## 17. Proteasome Activation by Small Molecules

### Highlighted by Michael Gütschow

Enhancing the activity of the ubiquitin-proteasome system (UPS) by increasing the ubiquitin pool or overexpressing specific ubiquitin ligases or improving the proteasome activity by low-molecular weight compounds can reduce the toxicity induced by protein aggregates and can be of therapeutic value in the treatment of neurodegenerative diseases. Huib Ovaa’s group, at the Netherlands Cancer Institute, together with several collaborators have used a proteasome activity probe in a high-throughput flow cytometry-based assay and identified compounds that increased proteasome activity, including the p38 MAPK inhibitor PD169316 [19]. Noteworthy, inhibition of p38 MAPK and its downstream target MK2 activates the proteasome. Thus, cellular players along the MAPK signaling cascade involved in proteasome activation have been discovered. To examine whether p38 inhibition can enhance the degradation of ubiquitinated proteins, a PROTAC method was employed showing that the cereblon-promoted degradation of BRD4 could be further augmented by preincubation with PD169316. By measuring the degradation of overexpressed α-synuclein in a bimolecular fluorescence complementation assay, the authors showed that inhibition of the p38 MAPK pathway and the subsequent activation of the proteasome represents a promising strategy to clear toxic protein aggregates. This study highlights the therapeutic potential of proteasome activation by distinct molecules. 

## 18. Secretome Analysis of BRCA1-Mutated Human Breast Cancer Reveals Novel Biomarkers

### Highlighted by Luigi A. Agrofoglio

The existence of a differential metabolic signature for breast cancer cells based on the BRCA1 mutation-associated tumors has been discovered by Spain researchers [20]. This approach, based on metabolomics, was to analyze and to characterize the metabolic profile of human breast cancer cell lines and plasma samples of triple-negative hereditary breast and ovarian cancer syndrome patients taking into account their BRCA1 genotype. 

It should be emphasized that one effective source for novel cancer biomarkers is the secretome, which contains molecules released by tumor cells into the extracellular space. The secretome could better reflect the activity of cancer in human body fluids such as blood plasma or serum.

It was shown that the levels of adenine, N6-methyladenosine and 1-methylguanine detected in the plasma samples of patients with BRCA1 mutations were significantly lower than those in patients who were not carriers of this mutation. 

It is believed that these biomarkers have a great potential to refine breast cancer diagnosis and enable personalized treatments.
